# A clinical perspective study on the compliance of surgical safety checklist in all surgical procedures done in operation theatres, in a teaching hospital, Ethiopia, 2021: A clinical perspective study

**DOI:** 10.1016/j.amsu.2021.102702

**Published:** 2021-08-10

**Authors:** Abraham Tarekegn Mersh, Debas Yaregal Melesse, Wubie Birlie Chekol

**Affiliations:** Department of Anaesthesia, College of Medicine and Health Sciences, University of Gondar, Gondar, Ethiopia

**Keywords:** Clinical perspective, Surgical safety checklist, Compliance

## Abstract

**Background:**

Patient safety during surgery is an important component for good outcome of operated patients. To discuss an important details about each surgical case; surgical safety checklist is an important patient safety tool that is used by the team of operating room professionals. This study aimed to identify the compliance of surgical safety checklist.

**Methods:**

This clinical perspective study was conducted from February 20 to March 20; 2021 at a teaching referral hospital. All surgical procedures done at a Comprehensive Specialized Teaching Hospital operation theatres were included. Data were collected through direct observation using World Health Organization standard checklist. Descriptive statistics were performed using SPSS version 20.

**Results:**

A total of 100 operations were observed in the main operation theatres of their surgical safety before induction of anaesthesia, before surgical incision and before any team member leave the operation room. From those 100 surgical procedures; patients’ identity, procedure and informed consent, anaesthesia machine checking and medication preparations were performed fully (100%) compliance with the standards.

**Conclusions:**

some standards weren't compliant with the standards of WHO surgical safety checklists. We recommend preparing common discussion panel for the operation room team about the performance of the surgical safety checklists and act accordingly.

## Highlights

• Patient safety during surgery is an important component.
• Patients' identity, procedure, informed consent were compliant with the standards.
• Some standards weren't compliant with the standards of surgical safety checklists.
• Risk of bleeding documentation and essential imaging displayed had zero compliant.

## 1 Introduction

Surgical care has been an important component of common health care worldwide for over a century. Because of the continuous increment in prevalence and incidences of traumatic injuries, cancers and cardiovascular disease, the impact of surgical intervention on public health systems will continue to grow and surgery became an integral part of global health care delivery [1,2]. Surgery is often the only therapy that can alleviate disabilities and reduce the risk of death from common conditions. Every year, many millions of people undergo surgical treatment, and surgical interventions account for an estimated 13% of the world's total disability-adjusted life years. On the other hand even though surgical procedures are intended to save lives, unsafe surgical care can cause substantial harm [3,4].

There are four underlying challenges to improving surgical safety. From those the first one is, not being considered as a significant public concern. It is assumed to be of limited relevance in poor and middle income countries due to high expense of surgical care. However the WHO global burden of disease report showed that a significant number of disabilities from disease in the world is due to conditions that can be treatable by surgical intervention. The second main problem in surgical safety has been a paucity of basic data. The data that were available were not standardized and varied widely in the types of procedures recorded. The third underlying problem has been that the existing safety practices do not appear to be used reliably in any country. Lack of resources is an issue in low-income settings, but it is not necessarily the most important one. The fourth underlying problem in improving surgical safety is its complexity [5,6]. The most critical resources of operating teams are the knowledge and experience of the clinicians (the surgeons, anaesthetists, nurses and others). A team should works effectively together to use its knowledge and abilities on behalf of the surgical patient to safe surgery. Yet, there are certain complexity between those professionals rather than working in cooperatively [7,8].

The main goal of the World Health Organization Patient Safety Checklist is for safe surgery and to saves lives and to improve the safety of surgical care around the world by defining a core set of safety standards that can be applied in all countries and settings [9]. A surgical safety checklist is a patient safety communication tool that is used by the teams of operating room professionals to discuss important details about each surgical case. It is a final check prior to surgery used to make sure everyone knows the important medical information they need to know about the patient, all equipment is available and in working order, and everyone is ready to proceed. This clinical perspective study was done to evaluate whether a Comprehensive Specialized Hospital Operation Theatres is performing safe surgery based on the already set safe surgery standards by World Health Organization or not.

## 2 Methods

This clinical perspective study was conducted from February 20 to March 20; 2021. All surgical procedures done at a Comprehensive Specialized Teaching Hospital in Ethiopia operation theatres were included. The data was collected through a direct observation using a standard check list of World Health Organization and by asking the team members after obtaining ethical approval from IRB with reference number of SOM/08/2021. Descriptive statistics were performed using SPSS version 20. The standards were directly changed into question forms with two integral checking components, “Yes”, and “No” (checklists)**.**

## 3 Results

A total of 100 operations were observed in the operation theatres from February 20 to March 20, 2021 of their surgical safety before induction of anaesthesia, before surgical incision and before any team member leave the operation room. From those 100 surgical procedures; patients’ identity, procedure and informed consent, anaesthesia machine checking and medication preparations are performed fully (100%) compliance with the standards. On the other hand; risk of bleeding documentation, specific concerns reports of surgeons VTE prophylaxis administration for those who are indicated, essential image displaying for patients with images were not applied for all procedures. As the table illustrates clearly, of the standards of before skin incision, above 75% of them were not implemented. However, of the standards of before any member of team leave the operating room, above 75% of them were appropriately implemented in the main operation theatres **(**Table 1**).**

**Table 1:** Frequency distribution of directly observed and interviewed techniques for safe surgery at a Comprehensive Specialized Teaching Hospital in Ethiopia operation theatres from February 20 to March 20/2021 (N = 100).

S.No.	Standards	Yes	%	No	%
Before induction of Anaesthesia					
1	Patients' identity, procedure and consent confirmed	100	100	0	0
2	Mark surgical site	56	56	44	44
3	Anaesthesia machine and medications checked	100	100	0	0
4	Known allergy checked	72	72	28	28
5	Difficult airway/aspiration checked	88	88	12	12
6	Risk of bleeding documented	0	0	100	100
Before surgical incision					
7	All team members introduced themselves by name and role	0	0	100	100
8	Surgeon, Anaesthetist and registered practitioner confirm verbally patient name, planned procedure, site and position	22	22	78	78
9	Surgeons blood loss estimation	28	28	72	72
10	Patient specific concern for surgeons checked	2	2	98	98
11	Patient specific concern for anaesthetist checked	22	22	78	78
12	Patients ASA grade checked	83	83	17	17
13	Nurses confirmation about the sterility of instrumentation	94	94	6	6
14	Antibiotic prophylaxis given	88	88	12	12
15	VTE prophylaxis given	0	0	100	100
16	Essential imaging displayed	0	0	100	100
Before any member of the team leaves the operating room					
17	Record the name of the procedure	88	88	12	12
18	Confirm instruments, swabs and sharps counts are complete	78	78	22	22
19	Specimens labeled by patient name	50	50	50	50
20	Any equipment problem need to addressed	12	12	88	88
21	Report key concerns for the recovery room professionals	56	56	44	44

### 3.1 Standards met before surgical incision

**Fig. 1:**
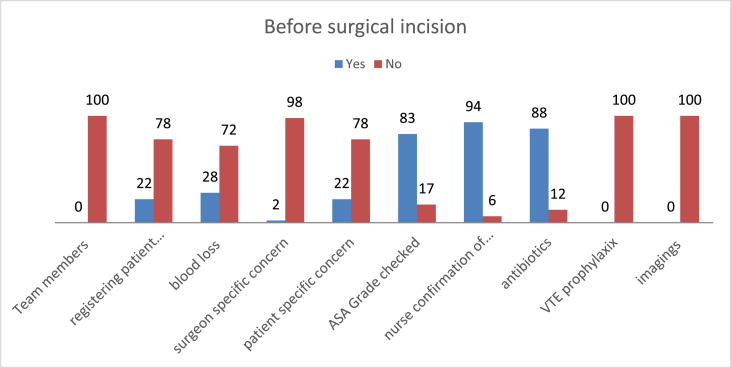
Standards met before surgical incision at a Comprehensive Specialized Teaching Hospital in Ethiopia operation theatres from February 20 to March 20/2021 (N = 100). Standards met before any member of the team leaves the operating room.

As indicated in Fig. 1 nearly 75% of the standards were below 50% and 33% of the standards were not met completely. On the other hand; patients ASA grade checking, nurses confirmation about the sterility of the instrumentation and antibiotic prophylaxis administration were performed nearly with the standard **(**Fig. 1**).**

**Fig. 2:**
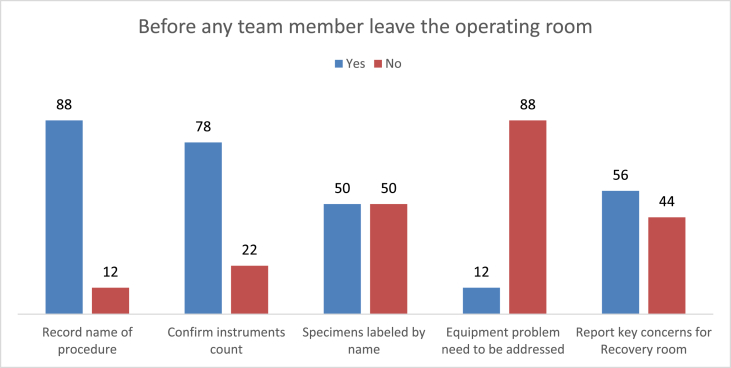
Standards met before any member of the team leaves the operating room at a Comprehensive Specialized Teaching Hospital in Ethiopia operation theatres from February 20 to March 20/2021 (N = 100).

As indicated in the below Fig. 2, 75% of techniques performed before any team leaving the OR were met the standards but, only four of equipment problems were addressed and 88% of the procedures were recorded **(**Fig. 2**).**

## 4 Discussion

As World Health Organization estimated that 234 million operations are performed annually around the globe. Of those procedures 9.2% were faced preventable harms daily during surgery across the world. These extra numbers, when extrapolated to the global number of surgeries conducted, are alarming and provide clear motivation to make surgery safer. That is why the World Health Organization surgical safety checklist was introduced as one means of reducing harm and improving patient safety in the operating theatre and also for better outcome of surgical patients. According to World Health Organization this checklist has been implemented around the world, and encourages dialogue within multidisciplinary teams and the use of routine safety checks to minimize harm to our patients [[10], [11], [12]].

As one of Federal Ministry of Health tertiary health institution the operation theatres perform many numbers of operations per day. According to World Health Organization draft for safe surgery the standard have three sections which should be strictly followed and applied.

Those are checking and setting of surgical preconditions before induction of anaesthesia, before surgical incision and before any member of the team leave the operation room [13,14].

According to this surgical safety checklist in a Comprehensive Specialized Teaching Hospital in Ethiopia operation theatres surgical procedures had 69% standard performance before induction of anaesthesia, very low or around 34% standard performance of before surgical incision and around 57% standard performance of before any member of the team leaves the operating room of overall performance. The overall performance in this clinical perspective study was 53%. This might be due to lack of training and poor attitude towards this surgical safety checklist. In clinical audit done at London, the performance of surgical safety checklist was 7.9% with 8.5% rates of early complications but after training the performance was 96.9% with 7.6% rate of early complications. Seventy seven thought that surgical safety checklist improved team communication [1,14].

In audit done at UK, surgical checklist implementation was optimized, regardless of the setting, when used as a tool in multifaceted cultural and organizational programs to strengthen patient safety [5]. But in this clinical perspective study there were certain techniques standards which were not completely practiced in some procedures such as risk of bleeding documentation before induction of anaesthesia, team members introducing each other, VTE prophylaxis administration for the indicated patients, displaying essential images for the available ones before surgical incision. It may be due to this introducing surgical checklists are not as straightforward as it seems, and requires leadership, flexibility, and teamwork in a different way to that which is currently practiced. The concept of using a checklist in surgical and anesthetic practice was believed that by routinely checking common safety issues, and by better team communication and dynamics, perioperative morbidity and mortality could be improved. The magnitude of improvement demonstrated by the World Health Organization pilot studies was surprising. These initial results have been confirmed by further detailed work demonstrating that surgical checklists, when properly implemented, can make a substantial difference to patient safety [3,14,15].

So according to World Health Organization and Federal Ministry of Health goals, this surgical safety checklist is the best tool for safe surgery and for better outcome of surgical patients and is momentous and minimizes surgical harms if practiced fully in the operating theatres.

### 4.1 Limitations of the study

This clinical practice study didn't show the factors that affect the adherence of the team against the standards and didn't avoid the hawthorn effect.

## 5 Conclusions and recommendations

Nearly half of the standards were below 50% implemented in the operation theatres. Of the standards of before skin incision, above 75% of them were not implemented. However, of the standards of before any member of team leave the operating room, above 75% of them were appropriately implemented in our setup. So, we recommended preparing common discussion panel for the operation room team about the performance of the surgical safety, creating awareness about surgical safety for the team by short term training and encouraging and following operation room team about their surgical safety performance during each procedure.

## Ethical approval

Ethical approval from IRB with reference number of SOM/08/2021.

## Consent

Participants were well informed and agreed with no benefit obtained.

## Please state any sources of funding for your research

Not funded

## Author contribution

This work was carried out in collaboration among all authors. A. T. Mersha and D.Y.Melesse contributed to the conception the review and interpreted the literatures based on the level of evidence and revised the manuscript. W.B. Chekol participate in reviewing preparation of the manuscript. Both authors participate in preparation and critical review of the manuscripts. In addition, all authors read and approved the manuscript.

## Registration of Research Studies

Name of the registry: researchregistry

Unique Identifying number or registration ID: researchregistry6978

Hyperlink to your specific registration (must be publicly accessible and will be checked): https://www.researchregistry.com/browse-the-registry#home/

## Guarantor

Abraham Tarekegn Mersha (A. T. Mersha), Debas Yaregal Melesse (D.Y. Melesse), Wubie Birlie Chekol (W.B. Chekol)
